# Introducing Deep Eutectic Solvents as a Water-Free Dyeing Medium for Poly (1,4-cYclohexane Dimethylene Isosorbide Terephthalate) PICT Nanofibers

**DOI:** 10.3390/polym13162594

**Published:** 2021-08-05

**Authors:** Nadir Hussain, Sadam Hussain, Mujahid Mehdi, Muzamil Khatri, Sana Ullah, Zeeshan Khatri, Lieva Van Langenhove, Ick Soo Kim

**Affiliations:** 1 Nano Fusion Technology Research Group, Division of Frontier Fibers, Institute for Fiber Engineering (IFES), Interdisciplinary Cluster for Cutting Edge Research (ICCER), Shinshu University, Tokida 3-15-1, Nagano Prefecture, Ueda 386-8567, Japan; engr.nadir712@hotmail.com (N.H.); muzamilkhatri@gmail.com (M.K.); sanamalik269@gmail.com (S.U.); 2 Center of Excellence in Nanotechnology and Materials, Mehran University of Engineering and Technology, Jamshoro 76060, Pakistan; sadam11te75@gmail.com (S.H.); mujahid11te83@gmail.com (M.M.); zeeshan.khatri@faculty.muet.edu.pk (Z.K.); 3 Centre for Textile Science and Engineering, Department of Materials, Textiles and Chemical Engineering, Technologiepark 907, Zwijnaarde, 9052 Gent, Belgium; lieva.vanlangenhove@ugent.be

**Keywords:** ultrasonic energy, dyeing, deep eutectic solvents, electrospinning, disperse dye

## Abstract

Water, one of the most priceless sources of life, is becoming dangerously threatened and contaminated due to population growth, industrial development, and climatic variations. The drainage of industrial, farming, and municipal sewage into drinking water sources pollutes the water. The textile processing industry is one of the major consumers of water. Herein, the idea of water-free dyeing of electrospun poly (1, 4-cyclohexane dimethylene isosorbide terephthalate) PICT nanofibers is proposed. For this, two different deep eutectic solvents (DE solvents) were introduced as an alternative to water for the dyeing of PICT nanofibers in order to develop a water-free dyeing medium. For this, C.I. disperse red 167 was used as a model dye to improve the aesthetic properties of PICT nanofibers. PICT nanofibers were dyed by conventional batch dyeing and ultrasonic dyeing methods to investigate the effect of the dyeing technique on color buildup characteristics. Dyeing conditions such as dyeing time, temperature and, dye-concentration were optimized. Morphological and chemical characterization observations revealed a smooth morphology of dyed and undyed PICT nanofibers. The ultrasonically dyed nanofibers showed higher color strength and increased tensile strength compared to conventionally dyed nanofibers. Further, the consumption of electrical and thermal energy was also calculated for both processes. The results confirmed that the ultrasonic dyeing method can save 58% on electrical energy and 25% on thermal energy as compared to conventional dyeing.

## 1. Introduction

Recently, the characteristics and synthesis of polyesters based on isosorbide are gaining more attention in terms of exploring potential applications. Poly (1,4-cyclohexane dimethylene isosorbide terephthalate) PICT is one of the bio-based polymers and is widely used in different applications because of its ease of production, eco-friendly character, green source of raw material (wheat, sugar, and corn), unique molecular structure, good transition temperature, and mechanical properties [1]. The unique characteristics of nanofibers such as a high surface area and fine diameter have increased their use in a number of applications such as biomedical, surgical, tissue engineering, filtration membranes, wearable conductives, and apparel applications [2,3,4,5,6,7,8].

For advanced apparel applications, the enhancement of the aesthetic properties of nanofibers is equally important as their functional characteristics [4,5]. Some conventionally dyed nanofibers such as the dyeing of polyester nanofibers with disperse dyes using the batch dyeing method [6], the dyeing of cellulose acetate nanofibers with disperse dyes using the pad-dry and bake-dry method [7], and polyurethane nanofibers dyed using the pad-dry-bake method [8], have been previously reported. However, problems such as lower color strength and lower color fastness remained a challenge.

Water, one of the most priceless sources of life, is becoming dangerously threatened and contaminated due to population growth, industrial development, and climatic variations. The drainage of industrial, farming, and municipal sewage into drinking water sources pollutes the water. The textile processing industry is one of the major consumers of water [9]. In the past two decades, new dyeing techniques have been developed by researchers to replace the water in textile processing. Several new dyeing techniques have low environmental impacts such as supercritical carbon dioxide dyeing, Air Dye VR technology, and non-aqueous solvent dyeing [10]. Solvent dyeing is a dyeing process that can be carried out in a non-aqueous phase. Deep eutectic solvents (DES) are considered green solvents and have been utilized by many scientists to solve different environmental problems [11,12]. Due to the biocompatible characteristics of DES, it is widely being considered as a green medium for various applications, including bioengineering, phase separation, biocatalysis, and synthesis for electrochemical and electropolishing has been introduced [13,14,15]. Thus, the aim of green chemistry is to plan an environmentally compassionate chemical process and synthetic strategies to minimize the utilization of toxic chemicals at any stage of the process in textile industries or research laboratories. Thus the DES, due to the presence of salts (Choline Chloride), may interact with CNF and enhance the color yield without causing any environmental hazards [12].

Recently textile industries have become very conscious about limiting the consumption of electrical energy during processing, especially in those processes which increase the production cost [9]. Many textile industries are using thermal energy for different processes which are costly in the field of textiles, therefore, there is a need for an efficient method to reduce the loss of thermal energy during processing, and herein an attempt has been made to save thermal energy in terms of the dyeing process [16,17]. Meanwhile, ultrasonic energy for dyeing has emerged as a suitable alternative to conventional dyeing because sonication prevents the dye from aggregating and increases the cavitation which increases mass transfer kinetics, and this enhances the coloration proficiency which consecutively reduces the energy consumption [18]. The ultrasonic dyeing is considered clean, and it gives a uniform effect at low temperatures and ultimately reduces costs. [19]. The most recent ultrasonic dyeing methods include the ultrasonic dyeing of cellulose nanofibers, nylon nanofibers, polyurethane nanofibers [20,21,22], and polyacrylonitrile nanofibers [23].

Herein, dye-ability disperse dye [Disperse dye, Foron Rubine S-GFL 150 (CI Disperse Red-167:1, high energy level) (Red-167:1) was assessed keeping in mind the better color strength and chemical compatibility of disperse dyes with a polyester-based substrate. PICT nanofibers were dyed conventionally and ultrasonically in order to compare the dyeing efficiency, electrical, and thermal energy consumption. The color strength and colorfastness of ultrasonically dyed samples and conventionally dyed samples were measured in order to measure the effect of both processes on color buildup. Samples were examined with SEM and FTIR for the physicochemical properties. Furthermore, tests of the shrinkage and tensile strength of samples were also carried out. Moreover, the electrical and thermal consumption for both processes was also calculated in order to assess the energy consumption for both processes.

## 2. Experimental Section

### 2.1. Materials

**Scheme 1:**
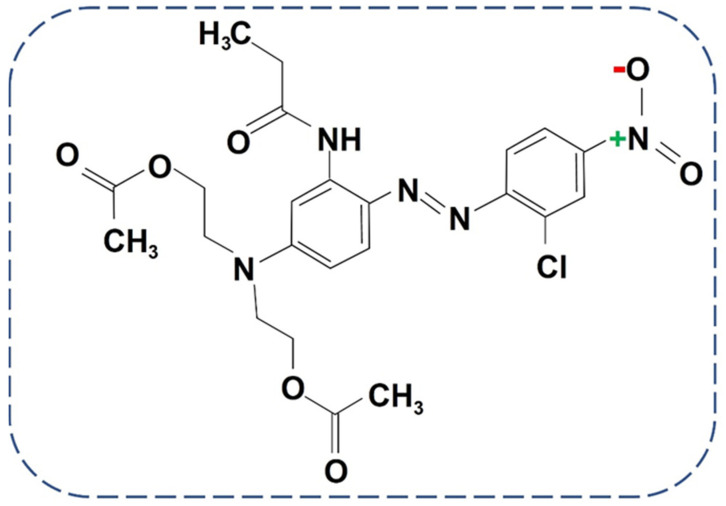
Chemical structure of C.I. Disperse Red-167:1, high energy level.

PICT with an average molecular-weight of M *n* ~ 26,000, Choline chloride (>98%) with a molecular weight of 139.62 g/mol, Furfuryl alcohol (98%) with a molecular weight of 98.10, Chloroform having a molecular weight of 119.38, and Trifluoroacetic acid (TFA) having a molecular weight of 114.02 were supplied by Sigma-Aldrich, USA. Ethylene Glycol with a molecular weight of 62.068 was purchased from Wako, Japan. Disperse dye, Foron Rubine S-GFL 150 (CI Disperse Red-167:1, high energy level) (Red-167:1) were obtained from Clariant (Pakistan). Scheme 1 shows the chemical structure of Red-167:1 dye.

### 2.2. Methods

#### 2.2.1. Preparing the PICT Nanofibers

PICT nanofibers were prepared using an electrospinning technique. The PICT polymer was dissolved in chloroform: TFA (3:1) and stirred for 2 h. In order to obtain bead-free nanofibers with stable electrospinning, PICT nanofibers were prepared using different polymer concentrations from 6% to 14% and optimized at 10% concentration. The applied voltage to the polymer solution was 10.6 kV using a high voltage power supplier (Har-100 × 12. Matsusada Co. Tokyo, Japan) for electrospinning, where the distance between tip and collector was kept at 16 cm. The nanofiber samples were pealed off after air drying for 24 h for further characterizations.

#### 2.2.2. Preparation of Deep Eutectic Solvents for Dyeing

Deep eutectic solvents have emerged as an alternate to the conventional organic solvents and ionic liquid to make organic transformations more efficient. Choline chloride, ethylene glycol, and furfuryl alcohol were the raw chemicals for the preparation of the deep eutectic solvents. Two types of deep eutectic solvents were prepared by alternating the mixing of choline chloride, ethylene glycol, and furfuryl alcohol. The first solvent was prepared by mixing choline chloride and furfuryl alcohol at 1:2, and the second solvent was prepared by mixing choline chloride and ethylene glycol at 1:2, and these solvents were named as CC:FF and CC:EG, respectively [23]. After mixing, both solutions were stirred at room temperature for 4 h for complete dissolution.

#### 2.2.3. Dyeing of PICT Nanofibers

Both conventional and ultrasonic methods were used for the batchwise dyeing of PICT nanofibers with disperse dye and two types of deep eutectic solvents used as a dyeing medium. Ultrasonic equipment (Model: elmasonic E3OH, elma Singen, Germany) with a fixed frequency of 38 kHz and output power of 320 W was used to carry out the ultrasonic dyeing of PICT nanofibers, and the intensity of the sonication bath was set at 0.98 W/cm2 (intensity = Power/bath area: 320–328). For dyeing, a 10 mg mass of PICT nanofibers having an average thickness of 120 µm was used. The ratio of dye liquor to the mass of nanofibers was maintained at 20:1. All samples were dyed by conventional and ultrasonic methods by optimizing at different dyeing parameters. The dyeing temperature was checked at (30 °C, 40 °C, 50 °C, 60 °C, 70 °C, and 80°C), dyeing time at (10, 20, 30, 40 and 50 min), and the dye concentration (2%, 3%, and 5%) were used to check color buildup of disperse dyes for dyeing PICT nanofibers. The results were optimized for both the conventional and ultrasonic dyeing of PICT nanofibers and compared with respect to dyeing efficiency and color strength.

#### 2.2.4. Color Spectrophotometry

The color strength of conventionally and ultrasonically dyed PICT nanofibers was measured using a data colors spectrophotometer and assessed by K/S values, calculated by Equation (1):

(1) $$K⁄S=\frac{\left(1-R\right)2}{2R}\times 100$$

where ‘R’ is a decimal fraction of the reflectance of dyed PICT nanofibers, ‘K’ denotes the absorption co-efficient, and ‘S’ is the scatting coefficient.

#### 2.2.5. Physical Morphology and Chemical Changes 

The changes in the chemical structure of dyed and undyed PICT nanofibers were assessed by the Fourier transform infrared spectroscopy on IR prestige—21 by Shimadzu Japan. All the dyed and undyed samples were analyzed by using ATR-FTIR mode. To analyze the physical structure, a scanning electron microscope model (JEOL JSM 6010LA), at a voltage of 30 kV with the highest magnification of 500,000× was used. The samples were sputtered by Pd-Pt before the morphology analysis.

#### 2.2.6. Color Fastness Tests

The color fastness test was carried out for checking the dyed PICT nanofibers. Color fastness to washing was performed in a gyro-wash (James H, Heal Co Halifax, UK), under the standard method of ISO 105, C10: 2006. The color fastness to light was also investigated according to ISO 105—B02 by Appollo (James, H Heal co. Halifax, UK)

#### 2.2.7. Tensile Strength Test

The tensile strength of prepared PICT nanofibers was investigated using the Universal Tensile Testing Machine (UTM, RTC-1250A, A&D Co., Ltd., Tokyo, Japan). The PICT nanofibers having a 120 µm average thickness were assessed, the tensile strength and tensile stress of all samples were compared, and Young’s modulus values were also calculated. [24]

#### 2.2.8. Shrinkage Test

A shrinkage test was performed by taking the PICT nanofiber sample size of 5 × 5 cm^2^ with a constant average thickness of 120 µm. The shrinkage of PICT nanofibers after dyeing was calculated by using Equations (4) and (5) for lengthwise shrinkage, and widthwise shrinkage, respectively.

(2) $$\mathrm{Ls}=\frac{L-\Delta L}{L}\times 100$$

(3) $$\mathrm{Ws}=\frac{W-\Delta W}{W}\times 100$$

(4) $$Rs=\sqrt{Ls^{2}+Ws^{2}}$$

In Equation (2), ***Ls*** is lengthwise shrinkage, ***L*** is the original length of the samples, **∆*L*** is the change in the original length after dyeing. In Equation (3), ***Ws*** is the widthwise shrinkage. ***W*** is the original width of samples. **∆*w*** is the change in the original width after dyeing. In Equation (4), ***Rs*** is the residual shrinkage after dyeing [5].

#### 2.2.9. Calculation of Electrical and Thermal Energy Consumption

The energy consumption of conventionally and ultrasonically dyed PICT nanofibers was calculated by the electrical energy Equation (5) as given below:

E = Pt(5)

where ‘E’ is energy (joule), ‘P’ denotes power (Watt), and ‘t’ is time (second).

Similarly, the thermal energy consumption of conventionally and ultrasonically dyed PICT nanofibers was calculated by the thermal energy Equation (6) as given below:

Q = m × c × ∆q (6)

where ‘Q’ is the amount of thermal energy (kcal), ‘m’ is the mass of liquor (gram), ‘c’ denotes the specific heat capacity of deep eutectic solvents, and ‘Δq’ is the change in temperature (°C).

## 3. Results and Discussion

### 3.1. Effect of Dyeing Temperature on PICT Nanofibers

**Figure 1:**
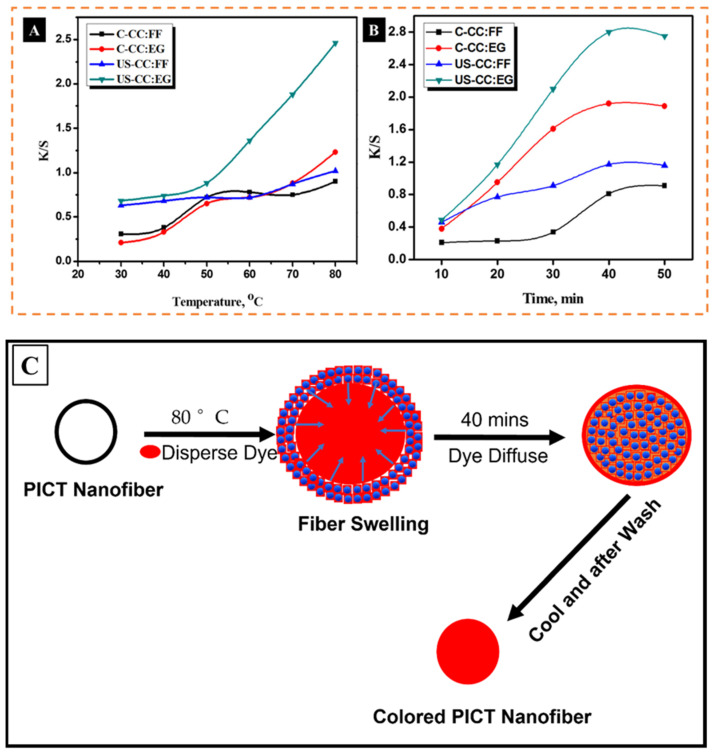
(**A**) Temperature optimization for ultrasonic dyeing of PICT nanofibers. (**B**) Time optimization for ultrasonic dyeing of PICT nanofibers. (**C**) Mechanism of dye diffusion.

PICT nanofibers were dyed with C.I. disperse Red-167:1 by ultrasonic and conventional methods at different temperatures. The dyeing temperature of PICT nanofibers was studied in the range of 30 °C to 80 °C by keeping the time constant at 30 min. Figure 1A shows the effect of dyeing temperature on the color strength of dyed nanofibers in both dyeing methods. The color strength of dye increased gradually with increasing dyeing temperature in CC:EG solvent from 30 °C to 50 °C and a rapid increase was observed from 50 °C to 80 °C, whereas in CC:FF solvent a slower increase in color strength from 30 °C to 60 °C and a rapid increase from 60 °C to 80 °C was observed. The higher color strength was observed in CC:EG solvent as compared to CC:FF solvent in both the ultrasonic and conventional methods, respectively. However, the ultrasonic dyeing in CC:EG solvent achieved a remarkable color strength. The ultrasonic dyeing of PICT nanofibers revealed higher color strength as compared to conventional dyeing. The high color strength of ultrasonically dyed samples is due to accelerated hydrolysis of deep eutectic solvents which increased the dye diffusion rate [25]. The mechanism of dye diffusion is explained in Figure 1C. Based on the above outcomes, 60 °C was chosen as an optimized temperature for both processes.

### 3.2. Effect of Dyeing Time on PICT Nanofibers

The time optimization for the dyeing of PICT nanofibers is just as important as temperature. At an optimized temperature, dyeing was carried out for different time intervals from 10 min to 50 min. Figure 1B shows that the dyeing in the CC:EG medium showed a notably higher increase in color strength from 10 min to 50 min in both dyeing methods, thus the maximum color strength was obtained at 40 min. The probable reason may be due to ultrasonication which effectively decreased the aggregation of dyes resulting in increased color strength [26,27]. The nanofibers dyed in the CC:FF medium also showed an increase in color strength from 10 min to 40 min with a gradual increase until 40 min. Hence, 40 min was chosen as an optimum time for dyeing PICT nanofibers with C.I. disperse Red-167:1 using ultrasonic energy. The ultrasonically dyed nanofibers presented better results as compared to conventional dyeing.

### 3.3. Effect of Dye Concentration on PICT Nanofibers

**Figure 2:**
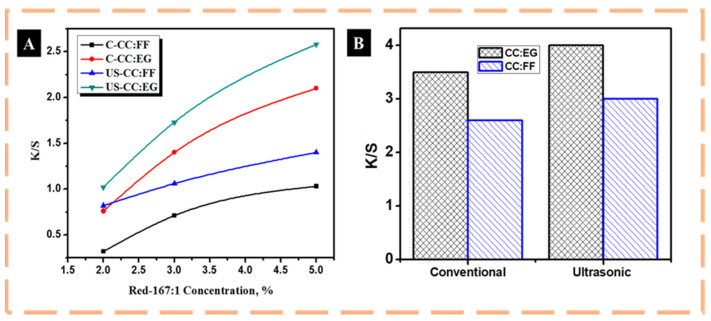
(**A**) Color buildup properties of Red-167:1 with respect to different concentrations. (**B**) Color build-up properties in the conventional, and ultrasonic methods.

Red disperse dye was used to dye PICT nanofibers in the CC:EG medium and CC:FF medium. The color build-up properties of PICT nanofibers as per dye concentrations are given in Figure 2A,B. The color build-up characteristics were compared using K/S values at three different dye concentrations (2%, 3%, and 5%) at a previously optimized time and temperature. Figure 2A,B shows the consistent increase in K/S values with increasing dye concentration for both media. The ultrasonically dyed PICT nanofibers showed a linear relationship that depicts very good dyeability and color build-up [17]. It can be observed in Figure 2A,B that the K/S values for dyeing PICT nanofiber in media CC:EG were higher than the PICT nanofiber in media CC:FF.

### 3.4. Comparison between Conventional Dyed PICT and Ultrasonic Dyed PICT with Respect to Color Strength

Ultrasonically and conventionally dyed PICT nanofibers have been compared with respect to color strength as shown in Figure 2B. The ultrasonically dyed PICT nanofibers showed 25% higher color strength than the conventionally dyed PICT nanofibers. In ultrasonic dyeing, nanofibers presented 25.9% higher results in the CC:EG medium than the CC:FF medium. The higher K/S value with ultrasonic dyeing may be attributed to the establishment of the resultant equilibrium for breaking dye aggregates in a short duration [28].

### 3.5. FTIR Spectra of Dyed and Undyed Nanofibers

**Figure 3:**
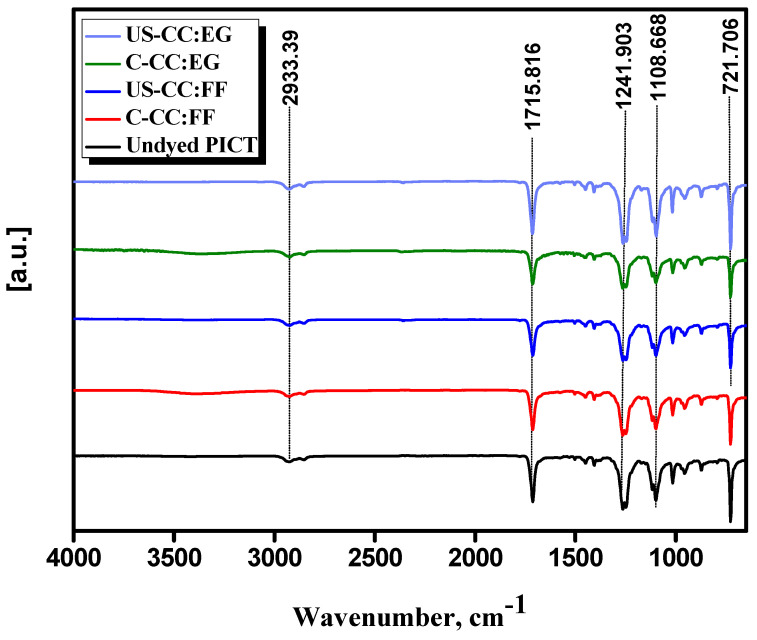
FTIR spectra of dyed and undyed PICT nanofibers using different dyeing methods and media.

An FTIR spectroscopy was carried out to examine the change in the chemical structure of the dyed and undyed PICT nanofibers shown in Figure 3. The stretching peaks at 1715 cm^−1^ and 1241 cm^−1^ confirms the presence of C=O and C-O in dyed and undyed PICT nanofibers. The spectra further showed the stretching of C-O at 1108 cm^−1^. The bending peak at 721 cm^−1^ confirms the presence of C=C in PICT nanofibers before and after dyeing. The functional groups of Red-167:1, CC:EG, and CC:FF could not be observed because a small number of respective chemicals were utilized to carry out the dyeing process and were rinsed well after dyeing [29]. Ultimately, it was confirmed by FTIR that PICT nanofibers can be dyed in the presence of deep eutectic solvents without altering the chemical structure of the PICT nanofibers.

### 3.6. Change in Crystallinity of PICT Nanofiber after Dyeing

**Figure 4:**
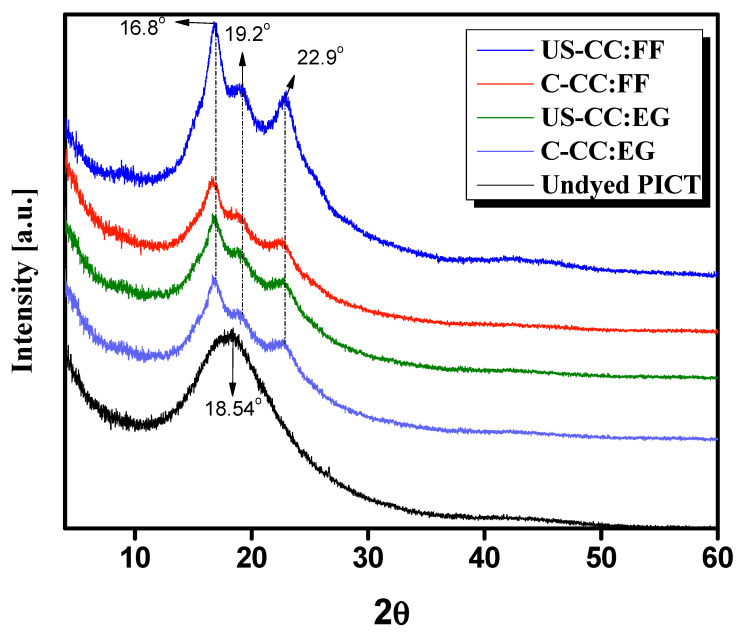
XRD patterns of dyed and undyed PICT nanofibers using different dyeing methods and media.

To analyze the PICT nanofiber’s properties, it is necessary to perform the XRD on dyed and undyed samples. Figure 4 shows the XRD patterns of undyed PICT nanofibers and PICT nanofibers dyed in different media of DE solvents. Undyed PICT shows intensive peaks at 18.5° with d-spacing (4.6 A°) and dyed PICT with CC:EG either conventionally or ultrasonically showed the same peaks at angles 16.8° and 22.9° with d-spacing (5.2 A°) and (3.7 A°), respectively. Whereas, conventionally dyed PICT with CC:FF shows peaks at the same angles as dyed the PICT in CC:EG but ultrasonically dyed PICT in CC:FF shows the peak at 16.8° with d-spacing (5.2 A°) but at angle 22.9° showed a more intensive peak with d-spacing (3.7 A°), respectively. The distance between the molecules in the chemical chain increases due to the decreasing diffraction angle in ultrasonically dyed samples but there were no effects found in the properties of PICT nanofibers [30].

### 3.7. Morphological Observation of Dyed and Undyed PICT Nanofibers

**Figure 5:**
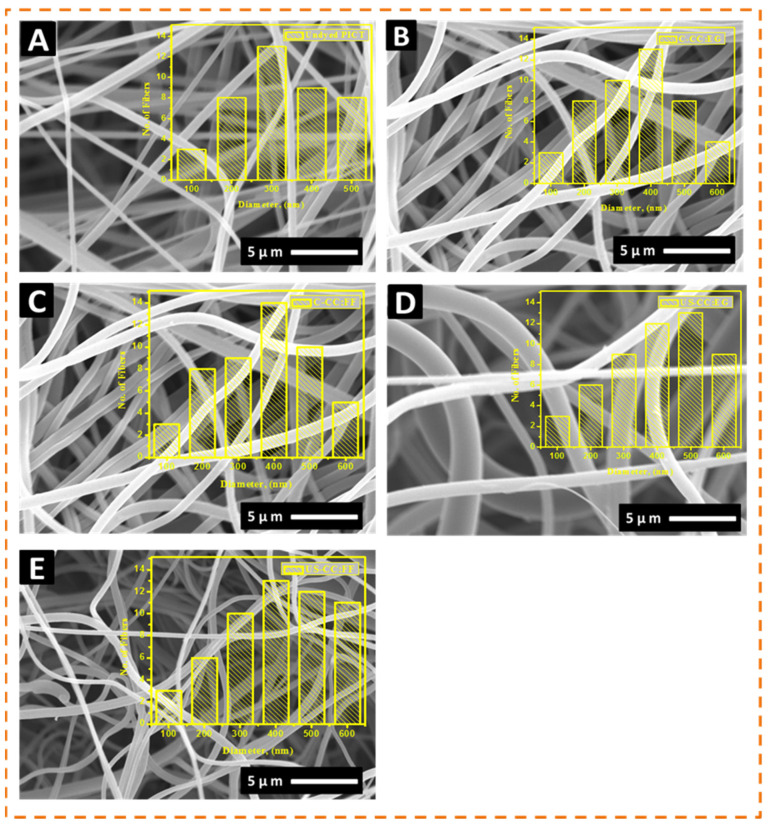
SEM image inside histogram of undyed PICT nanofibers (**A**), PICT nanofibers conventional dyed in CC: EG (**B**) and dyed in CC: FF (**C**), PICT nanofibers ultrasonically dyed with CC: EG (**D**) and CC: FF (**E**).

Figure 5 shows the smooth morphology of dyed and undyed PICT nanofibers. Figure 5A shows the smooth morphology of undyed PICT nanofibers and Figure 5B,C show the SEM image of conventionally dyed PICT nanofibers in CC:EG and CC:FF. In Figure 5D,E ultrasonically dyed PICT nanofibers in CC:EG and CC:FF can be seen with smooth morphology where the ultrasonic dyeing of PICT nanofibers shows a small increase in diameter. Similarly, the smooth morphology of conventionally dyed PICT nanofibers in CC:FF and ultrasonically dyed PICT nanofibers also show smooth morphology but with increasing diameter. Although, no change could be observed between conventionally dyed PICT nanofibers in both mediums and undyed PICT nanofibers, the diameter of ultrasonically dyed PICT nanofibers in both media CC:EG and CC:FF was observed as slightly bigger than undyed PICT nanofibers as shown in Figure 5B,D. Shrinkage during the ultrasonic dyeing of PICT nanofibers was the reason for small increases in nanofiber diameter.

### 3.8. Color Fastness Performance of Nanofibers

The color fastness test was performed on the dyed PICT nanofibers which are presented in Table 1. The staining in washing fastness for Red-167:1 has attained a maximum rating in both conventionally and ultrasonically dyed PICT nanofibers in CC:EG. Where the Red-167:1 showed slightly lesser in both ultrasonically and conventionally dyed nanofibers in CC:FF which indicates good dye fixation properties for both dyes and media. The changes in color fastness showed very good ratings, 4/4 and 4/5 for both dyes irrespective of the dyeing medium. Ultimately ultrasonically dyed PICT nanofibers averagely showed better dye fixation in the washing fastness test. The conventionally and ultrasonically dyed PICT nanofibers dyed with respect to dye type and medium showed no difference in the light fastness test results and achieved an average in all dyed samples.

### 3.9. Shrinkage in PICT Nanofibers after Ultrasonic Dyeing

PICT nanofibers after exposure to sonication showed substantial shrinkage so it was necessary to analyze the shrinkage in PICT nanofibers after dyeing. In Table 2 there is an emphasis on documenting the findings. It is known by default that the article should report a new finding in order to affirm its originality and to become worthy of publication. The shrinkage results were also dependent on the medium used, irrespective of the dyeing method. In CC:EG the shrinkage percentage is higher than in CC:FF. Ultrasonication resulted in maximum shrinkage within PICT nanofibers after dyeing, ultimately, shrinkage was also the reason for higher color strength values compared to the conventional dyeing of PICT nanofibers.

### 3.10. Young’s Modulus Values of Dyed and Undyed PICT Nanofibers

**Figure 6:**
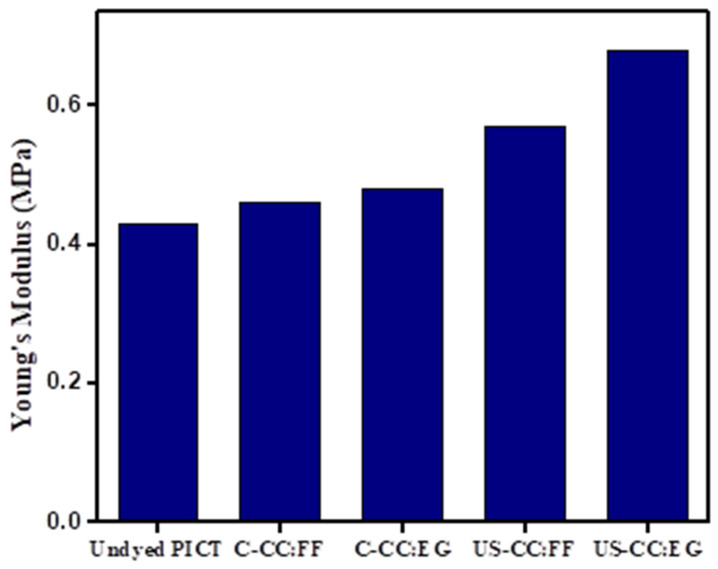
Young’s Modulus values of dyed and undyed PICT nanofibers using different dyeing methods and media.

Figure 6 shows the Young’s modulus values of dyed and undyed PICT nanofibers. Undyed PICT nanofibers showed the lowest Young’s modulus up to 4.3 MPa compared to all dyed PICT samples. The incorporation of dye within the PICT nanofiber was the result of an increasing Young’s modulus. Irrespective of dye incorporation, the PICT nanofibers dyed in CC:EG showed slightly higher Young’s modulus values than PICT nanofibers dyed in CC:FF. Regardless of the medium and dye incorporation, ultrasonically dyed PICT nanofibers showed higher Young’s modulus values than the conventionally dyed PICT nanofibers, which may be due to more shrinkage in ultrasonically dyed PICT nanofiber samples.

### 3.11. Electrical and Thermal Energy Consumption for Dyeing PICT Nanofiber

For the dyeing of PICT nanofibers, electrical energy consumption was compared between the conventional dyeing method and ultrasonic dyeing method as given in Table 3A. Keeping time and temperature in consideration, the electrical energy was calculated in joules per second for both conventional and ultrasonic dyeing methods. The results showed that both DE solvents have similar energy consumption using the conventional dyeing method. While the ultrasonic dyeing method can save 58.75% more electrical energy in the CC:EG medium compared to conventional dyeing, and ultrasonic dyeing in CC:FF can save 56.8% more electrical energy compared to conventional dyeing in the CC:FF medium.

The thermal energy was also compared between the ultrasonic dyeing and conventional dyeing method as shown in Table 3B. Ultrasonic dyeing in the CC:EG medium saves 25% more thermal energy compared to the conventional dyeing in the CC:EG medium, and ultrasonic dyeing with CC:FF saves 25.24% more energy compared to conventional dyeing in the CC:FF medium. Hence, dyeing using ultrasonic energy is superior not only in terms of enhanced efficiency and better results of dyeing, but it saves electrical and thermal energies as well.

## 4. Conclusions

The PICT nanofibers were successfully dyed in DE solvents with disperse dye using ultrasonic and conventional dyeing methods. Physiochemical outcomes confirmed the presence of dye molecules on the fiber surface without altering the chemical structure of the electrospun PICT nanofibers. Colorimetric studies and colorfastness tests suggest better color buildup properties of the ultrasonic-assisted dyeing of PICT nanofibers with DE solvent than that of the conventional dyeing method. It was found that shrinkage was the ultimate reason for increased color strength in ultrasonically dyed PICT nanofibers. Further, Ultrasonic dyeing in DE solvents proved to be a better choice for environmentally friendly and cleaner production, this cleaner approach can save electrical energy (58.6 and 58.7%) and thermal energy (25 and 25.4%) with CC:FF and CC:EG mediums, respectively. The crystallinity and tensile strength were increased in PICT nanofibers after dyeing.

## Figures and Tables

**Table 1:** Color fastness properties of dyed PICT nanofibers in DE solvents.

Dye Class	Method	Light Fastness (20 h)	Washing Fastness	Washing Fastness (Staining on Different Fibers)					
				CT	CO	PA	PES	PAC	WO
Red-167:1	C-CC:EG	2	4	4/3	4/5	3	4	5	5
	US-CC:EG	2	4/3	4/5	5	3/4	4	5	5
	C-CC:FF	2	4	4	4/5	2/3	3/4	5	5
	US-CC:FF	2	4/5	5	4/5	3/4	4	5	4/5

^a^ Dyeing parameters: dyeing temperature 60 °C and dyeing time 40 min. ^b^ CT, cellulose triacetate; CO, cotton; PA, polyamide; PES, polyester; PAC, polyacrylic; Wo, wool.

**Table 2:** Shrinkage in PICT nanofibers after conventional and ultrasonic dyeing.

Shrinkage	Conventional Dyeing		Ultrasonic Dyeing	
	CC:FF	CC:EG	CC:FF	CC:EG
Lengthwise	13%	16%	18%	23%
Widthwise	17%	19%	21%	26%

^a^ Shrinkage study was calculated by using Equations (2) and (3).

**Table 3:** (**A**). Electrical energy consumption of PICT nanofibers after conventional and ultrasonic dyeing. (**B**). Thermal energy consumption of PICT nanofibers after conventional and ultrasonic dyeing.

(A) Method	Temperature (°C)	Time (min)	Total Consumption (Joules Per Second J/sec)	Saving Energy (%)
C-CC:EG US-CC:EG	80	50	480,000	58.75
	60	40	81,000	
C-CC:FF US-CC:FF	80	60	480,000	56.6
**(B)** **Method**	**Temperature (°C)**	**Mass of Liquor in g**	**Total Consumption/(Joules Per Second J/sec)**	**Saving Energy (in %)**
US-CC:EG	80	0.01	82.86	25.4
C-CC:EG	80	0.01	110.48	
US-CC: FF	60	0.01	187	25
C-CC: FF	60	0.04	250.16	

^a^ Electrical energy was calculated using Equation (5). ^b^ Thermal energy was calculating using Equation (6).

## Data Availability

The data can be requested from the corresponding author of the article.
